# Pathogenesis, Diagnosis, and Management of Trigeminal Neuralgia: A Narrative Review

**DOI:** 10.3390/jcm14020528

**Published:** 2025-01-15

**Authors:** Yao Liu, Eiji Tanaka

**Affiliations:** 1 Stomatological Hospital and Dental School of Tongji University, Shanghai Engineering Research Center of Tooth Restoration and Regeneration, Shanghai 200072, China; 22310021@tongji.edu.cn; 2 Department of Orthodontics and Dentofacial Orthopedics, Tokushima University Graduate School of Biomedical Sciences, Tokushima 770-8504, Japan

**Keywords:** trigeminal nerve, neuralgia, low-intensity pulsed ultrasound, management

## Abstract

Trigeminal neuralgia (TN) is an excruciating neurological disorder characterized by intense, stimulus-induced, and transient facial stabbing pain. The classification of TN has changed as a result of new discoveries in the last decade regarding its symptomatology, pathogenesis, and management. Because different types of facial pain have different clinical therapy and neuroimaging interpretations, a precise diagnosis is essential. Diagnosis should include magnetic resonance imaging with specific sequences to rule out secondary causes and to identify possible neurovascular contact. The purpose of demonstrating a neurovascular contact is to aid in surgical decision making, not to validate a diagnosis. Microvascular decompression is the first-line procedure for individuals who do not respond to medical management, whereas carbamazepine and oxcarbazepine are the preferred medications for long-term care. New developments in animal models and neuroimaging methods will shed more light on the biology and etiology of TN. This paper reviews the pathogenesis, the clinical features, the diagnosis, and the management of TN. Furthermore, the potential role of low-intensity pulsed ultrasound in neurological disorders is discussed.

## 1. Introduction

Trigeminal neuralgia (TN) is a debilitating neuropathic pain condition that significantly impairs the quality of life of sufferers, affecting not only their physical but also their psychological and social conditions. The intense, episodic, and shock-like facial pain characteristic of TN disrupts daily activities, and is underscored by a demonstrable increase in anxiety, depression, and compromised sleep quality among sufferers [1]. The multifaceted impact of TN necessitates a holistic understanding and approach to its management, involving medical, dental, and surgical healthcare professionals [2]. However, the diversity of treatment approaches underscores the need for a unified evidence-based perspective, especially considering the recent advances in our understanding of the symptomatology, etiology, pathophysiology, classification, and treatment remedies for TN.

Recent studies have highlighted significant advances in the diagnostic imaging of TN, with conventional magnetic resonance imaging (MRI) revealing neurovascular contact (NVC) at the trigeminal nerve root entry zone, which correlates closely with the symptomatic side of TN [3,4,5]. This neurovascular interaction is thought to play a pivotal role in the manifestation of TN symptoms, leading to a re-evaluation of previous classification systems [3,4,5]. Beyond the anatomical changes observed in conventional imaging, sophisticated techniques such as diffusion tensor imaging (DTI) have highlighted changes in the microstructure of trigeminal nerve myelination, attributing the pathophysiology to either dysmyelination or demyelination [6]. These findings not only enhance our understanding of the underlying mechanisms of the disease, but also refine its classification, which is now endorsed by leading neurological and pain research societies.

Epidemiologically, TN is a complex interaction of risk factors ranging from genetic predisposition to environmental triggers [7,8]. Advanced analytical methods such as Mendelian randomization have identified certain modifiable risk factors, including years of schooling, sleep duration, anxiety disorders, depression, autoimmune diseases, and body mass index, for the incidence of TN [9]. This multivariate analysis further complicates the landscape of TN by integrating it into a wider context of systemic health and lifestyle factors, emphasizing the complexity of its management. Clinical management of TN has traditionally oscillated between pharmaceutical, surgical, and, more recently, interventional neuroradiological treatments, aimed at alleviating the excruciating pain associated with the condition. Innovations in surgical techniques, including percutaneous procedures, offer promising outcomes in terms of pain relief and the alleviation of the psychological burden of the disease [10]. The assessment of such interventions, alongside the empirical evaluation of the burden of TN within specialist centers, provides a valuable framework for measuring treatment outcomes, patient satisfaction, and quality of life post-interventions [11]. This reflects an evolving recognition of TN as a multidimensional disorder that requires a holistic approach to its management, encapsulating physical, psychological, and social domains.

In exploring the therapeutic potential of low-intensity pulsed ultrasound (LIPUS), considerable interest has been generated due to its minimal thermal and notable non-thermal effects, suggesting profound therapeutic changes in tissues [12]. LIPUS diverges from traditional ultrasound by prioritizing therapeutic application over diagnostic imaging, making it a promising tool in rehabilitation medicine. The intrigue surrounding LIPUS lies largely in its reported ability to facilitate cell proliferation and multilineage differentiation, particularly of mesenchymal stem and progenitor cells (MSCs), suggesting its utility in clinical procedures aimed at neuropathy treatment, among other conditions. The research by Kusuyama et al. [13] provides significant insights into the molecular pathways activated by LIPUS, demonstrating that this modality can suppress adipogenesis while enhancing osteogenesis in MSCs through the Rho-associated kinase-Cot/Tpl2-MEK-ERK signaling pathway. This finding is pivotal as it is consistent with the therapeutic objectives of enhancing nerve regeneration and alleviating pain, and provides insight into the mechanistic underpinnings by which LIPUS elicits its biological effects [13].

Here we review the pathogenesis, diagnosis, and management of TN. Additionally, the potential role of LIPUS treatment for neurological disorders is discussed, providing a good-as-new management of TN.

## 2. Search Strategy and Study Selection

The electronic database Pubmed was searched to retrieve relevant articles published between 1992 and 2024 using the following terms “trigeminal neuralgia” AND “pathogenesis OR clinical features OR diagnosis OR management”; “low-intensity pulsed ultrasound” AND “neuroscience OR pain OR neuropathy OR nerve”. One of the authors (YL) screened all of the abstracts. Inclusion criteria were original articles, review articles, and case reports with a precise definition of the TN, published in English. Relevant publications were retrieved from the reference list and further analyzed to determine whether they met the inclusion criteria. The authors performed data retrieval and the quality and bias of the retrieved articles were not interpreted.

## 3. Pathogenesis of TN

A review of the literature on the symptomatology of TN, regardless of its etiology—be it classical, idiopathic, or secondary—highlights a convergence of evidence pointing to neural pathology at the root entry zone, primarily due to its compression by a blood vessel or a tumor [14,15,16]. This region is anatomically distinct because of the transition from peripheral Schwann cell myelination to central oligodendroglia myelination [17], which is thought to make it particularly vulnerable to compression. This susceptibility is further evidenced by biopsy specimens from surgery in the compressed region showing evidence of demyelination, demyelination, and remyelination, alongside direct apposition of demyelinated axons [18,19]. Such demyelinated afferents are known to become hyperexcitable, a phenomenon that leads to the generation of ectopic impulses and manifests as spontaneous pain [20]. Furthermore, it has been hypothesized that ephaptic connections between demyelinated Aβ and Aδ fibers may be responsible for touch-evoked pain [21], with the intense near-explosive characteristic pain possibly resulting from trigeminal ganglion cell somata developing touch-evoked prolonged discharges that propagate from cell to cell [20]. Support for the causative role of neurovascular compression at the root entry zone in TN comes from neurophysiological studies using scalp far-field evoked potentials and quantitative sensory testing (QST), both of which have shown normalization after microvascular decompression [22,23]. In idiopathic trigeminal neuralgia, suspected pathologies range from neuronal voltage-gated ion channel mutations leading to a gain of function to non-specific inflammation and non-multiple sclerosis brainstem lesions [24,25,26,27,28,29]. In this domain, a systematic clinical study using a genomic screen revealed that familial occurrences of TN, potentially linked to variants in genes encoding for voltage-gated ion channels, might be more common than previously considered [16]. These gene variants may be important in elucidating the pathogenesis of TN and underscore the genetic underpinnings of idiopathic cases. The presence of continuous pain, alongside paroxysmal pain in TN patients, has led some researchers to speculate about the role of centrally mediated facilitation of nociceptive processing or a decrease in descending inhibitory mechanisms [30,31]. In patients with both continuous and paroxysmal pain, greater nociceptive blink reflexes and increased brainstem-evoked potentials have been observed in addition to diminished conditioned pain modulation. However, a blinded QST study found it difficult to distinguish between the two patient groups, indicating the complexity of TN symptomatology and the need for further investigation [32]. Taken together, it is clear that the neuropathological features of TN, from the susceptibility of the root entry zone to neurovascular compression to genetic predisposition and central nervous system (CNS) involvement, paint a complex picture of this condition. The interplay of peripheral and central mechanisms, in addition to genetic factors, underscores the multifaceted nature of TN and requires a holistic approach to both research and treatment strategies to better understand and manage this debilitating condition.

## 4. Clinical Features of TN

The pain associated with TN is intense, sharp, and often described as an electric shock-like sensation [1]. Understanding the characteristics, causes, diagnostic criteria, and treatments for TN requires a thorough examination of the current research and literature in the field. The clinical features of TN have been well documented, revealing that patients typically experience short-lasting, stabbing, or electrical shock-like pain, often triggered by innocuous mechanical stimuli such as light touch, talking, or chewing [2,33]. Such detailed clinical descriptions are crucial for the accurate diagnosis and understanding the impact of TN on patients’ quality of life. Furthermore, studies have highlighted the heterogeneous nature of TN pain triggers, with some patients reporting atypical triggers such as weather changes or certain foods, suggesting the involvement of A-delta sensory afferents [34]. This variability in pain and triggers points to the complex pathophysiology underlying TN, which is still not fully understood. Effective diagnosis and management of TN depends heavily on distinguishing it from other types of facial pain and understanding its pathophysiology. For instance, the presence of neurovascular compression (NVC) at the nerve root entry zone is often implicated in classic TN, reflecting the importance of radiographic evidence in diagnosis [35]. Advances in imaging techniques and criteria, such as high sensitivity MRI for excluding central lesions and consideration of T2 signal intensity changes, have improved the diagnosis and understanding of the causes of TN [33,36]. Furthermore, the misdiagnosis of TN highlights the need for comprehensive clinical evaluation to ensure accurate diagnosis and appropriate treatment [37]. There is a strong case for multifaceted therapeutic approaches given the range of symptoms experienced by patients, including both intense paroxysmal pain and less severe but continuous dull pain. Recent exploration into pulsed radio frequency (PRF) stimulation presents an intriguing alternative, with promising results in alleviating TN pain with sustained effectiveness [38]. Such innovations in treatment highlight the ongoing efforts to improve the quality of life for TN patients by providing more effective and durable pain relief solutions. Additionally, understanding the broader impacts of TN, including quality of life issues and psychological aspects such as anxiety, is crucial for holistic patient care [2]. The association between TN and autoimmune diseases further exemplifies the need for a comprehensive approach to patient assessment and treatment planning, as these patients may exhibit different clinical characteristics and postoperative outcomes [39]. In summary, the current state of research on TN provides valuable insights into its clinical features, diagnostic criteria, and treatment options. Advances in diagnostic imaging and treatment, including PRF stimulation and consideration of comorbidities, are enhancing patient care. However, challenges in diagnosis and variable treatment outcomes underline the need for ongoing research and a nuanced understanding of the complexities of TN.

## 5. Diagnosis of TN

TN is a debilitating facial pain condition that has long challenged the medical community in terms of accurate diagnosis and effective treatment. The complexity of this condition lies not only in its symptomatic presentation but also in the intricate diagnostic process required to distinguish it from other facial pain syndromes [40,41]. The literature abundantly reflects the multifaceted approach required to understand, diagnose, and manage TN. The diagnosis of TN is primarily based on the patient’s clinical history and a detailed examination to rule out other causes of facial pain. This approach is indispensable, as neuroimaging and other diagnostic tests, although helpful, are not definitive for TN on their own. Sensory abnormalities, primarily mild hypoesthesia, have been frequently reported in TN patients, even in those without previous surgical intervention [42].

TN has three classifications: classical trigeminal neuralgia, secondary trigeminal neuralgia, and idiopathic trigeminal neuralgia. The diagnostic criteria for classical trigeminal neuralgia are recurrent paroxysms of unilateral facial pain and demonstration on MRI or during surgery of neurovascular compression (not simply contact) with morphological changes in the trigeminal nerve root. For secondary trigeminal neuralgia, the diagnostic criteria are recurrent paroxysms of unilateral facial pain fulfilling TN criteria, either purely paroxysmal or associated with concomitant continuous or near-continuous pain; an underlying disease has been demonstrated that is known to be able to cause, and explain, the neuralgia; not better explained by another ICHD-3 diagnosis. Idiopathic trigeminal neuralgia is recurrent paroxysms of unilateral facial pain fulfilling criteria for TN, either purely paroxysmal or associated with concomitant continuous or near-continuous pain; neither classical trigeminal neuralgia nor secondary trigeminal neuralgia have been confirmed by adequate investigation, including electrophysiological tests and MRI; not better accounted for by another ICHD-3 diagnosis [43].

In contrast with TN, other types of facial pain, such as toothache, tend to be more persistent, and the characteristics of the pain and the triggers vary significantly due to their different causes. For example, pulpitis is often associated with a persistent dull pain rather than short bursts of intense pain [44]. Another important aspect of identifying TN is understanding the underlying cause of the pain. Classical TN is usually caused by vascular compression of the trigeminal nerve root, while secondary trigeminal neuralgia may arise from other diseases such as tumors or multiple sclerosis. Therefore, during the clinical evaluation, physicians should often perform detailed imaging studies, such as MRI, to exclude possible secondary factors [45,46]. Comparing these different types of pain, following specific clinical diagnostic criteria and guidelines can help to identify the characteristics of pain more quickly and accurately [47].

A Danish study highlighted this clinical challenge, revealing that a significant proportion of patients had these sensory distortions [48]. The subtlety of these abnormalities necessitates a keen clinical sense to prompt further investigation, especially when the neurological examination is largely normal, which is the case in many individuals diagnosed with TN. QST has been instrumental in unveiling the nuanced sensory deficits in TN. A pivotal blinded QST study demonstrated a general increase in the mechanical detection thresholds on both the symptomatic and asymptomatic sides of the face and hands of patients with TN [21,32,49,50]. These findings extend the understanding of TN beyond the traditional view of a peripheral nerve disorder and suggest a role for pain-induced central somatosensory plasticity. This notion is further supported by the presence of hypoesthesia and hyperalgesia, adding complexity to the pathophysiological understanding of TN. There is evidence that these sensory abnormalities may also indicate central mechanisms of pain sensitization, bridging the gap between peripheral trigeminal nerve dysfunction and central nervous system involvement [32]. The differential diagnosis of TN includes a wide array of conditions, emphasizing the need for a thorough clinical evaluation to facilitate appropriate referral [51]. In addition, the overdiagnosis of TN, primarily due to an overreliance on neuroimaging findings such as MRI-detected NVCs, has been identified as a common pitfall. This overdiagnosis is generally due to insufficient clinical assessment, highlighting the critical role of a comprehensive history and physical examination in differentiating TN from other facial pain disorders [37]. In addition to the conventional diagnostic approaches, novel techniques and criteria are continually explored to improve the accuracy and reliability of TN diagnosis. Despite the evolving landscape of diagnostic strategies, the primary reliance on clinical judgment based on patient history and clinical examination remains the cornerstone of TN diagnosis. Collectively, these studies underscore the complexity of diagnosing TN, which requires a multidimensional assessment that integrates clinical acuity with advanced diagnostic tools.

## 6. Management of TN

### 6.1. Acute Intervention for Severe Exacerbations

The management of acute exacerbations of TN is a major challenge in clinical practice due to the sudden and severe nature of the pain episodes [52]. In the absence of randomized controlled trials to guide acute medical treatment, clinicians often rely on a combination of clinical experience and evidence from retrospective studies and case series [52,53]. A retrospective analysis of 144 cases by Muñoz-Vendrell et al. [54] found that intravenous lacosamide and phenytoin can be effective and safe treatments for acute pain in TN, suggesting that lacosamide may be better tolerated than phenytoin, leading to lower readmission rates and sustained pain relief. This finding is particularly relevant given the description’s emphasis on the need for effective in-hospital treatments for severe TN exacerbations, which might lead to dehydration and anorexia. The ability of these medications to provide rapid pain relief could be crucial in stabilizing patients until more definitive treatments can be arranged. Another retrospective study explored the immediate pain relief achieved by lidocaine aerosol sprayed onto the oral and/or nasal mucosa in patients suffering from acute TN exacerbations. The study suggests that this approach may be a promising treatment option, consistent with the clinical view that lidocaine injections into trigger areas may provide short-term relief [55]. Given the description’s reference to the potential benefits of lidocaine for acute pain relief, this study supports the trend towards the use of local anesthetics as a part of acute management strategies. The efficacy of low-dose fosphenytoin in relieving acute TN pain was the focus of a case report [56], underscoring the viability of fosphenytoin for pain relief in the emergency department setting. This is in line with the demand for effective acute management strategies in high dependency units as described. Although the evidence comes from a single case, it reinforces the clinical utility of fosphenytoin, alongside lidocaine, for immediate pain control. The role of phenytoin in relieving acute TN exacerbations was further supported by a retrospective case series [57], which demonstrated the safety and effectiveness of the drug. This evidence base, although derived from retrospective analyses, fills the gap in randomized controlled trials for the management of acute TN and supports the use of intravenous antiepileptic drugs as proposed in the description. In conclusion, the management of acute TN exacerbations remains a complex challenge due to the lack of high-level evidence from randomized controlled trials. However, retrospective studies and case reports provide valuable insights into potential treatment strategies. Intravenous lacosamide, phenytoin, and the innovative use of lidocaine aerosol [54,55] are emerging as practical options for providing immediate pain relief in the acute setting. As the condition requires rapid and effective intervention to prevent complications such as dehydration and anorexia, these findings underscore the importance of exploring all viable options for acute pain management in TN. Further research, ideally in the form of randomized controlled trials, is needed to consolidate these treatment strategies within standardized clinical guidelines.

### 6.2. Prolonged Pharmacological Therapy

The quest for effective long-term pharmacological treatment options for TN reflects the complexity and severity of this condition. Despite the high prevalence of TN and its impact on patients’ quality of life, the evidence to guide management strategies, especially in terms of pharmacological interventions, remains markedly sparse. The literature indicates a strong preference for antiepileptic drugs, particularly carbamazepine and oxcarbazepine, as the first-line management therapy for TN pain [58,59]. These medications have demonstrated efficacy in reducing the neuropathic pain associated with TN; however, their use is often limited by adverse side effects. This dilemma underscores the need for continuous monitoring and careful dose adjustment to maintain a balance between pain relief and tolerability. The inadequacy of carbamazepine and oxcarbazepine due to tolerability issues or adverse effects has led clinicians and researchers to explore alternative or adjunctive pharmacological options. Gabapentin and nortriptyline represent such alternatives, although their comparative efficacy and acceptability require further clarification through rigorous evaluation [59,60]. Furthermore, the recent consideration of botulinum toxin type A as an adjunctive or alternative treatment modality signals a progressive shift towards exploring non-traditional pharmacological interventions for TN. This development suggests a promising avenue for future research, particularly in light of the call for large-scale randomized controlled trials to validate its efficacy and safety profile [59]. However, the analysis extends beyond efficacy and safety profiles to consider the utility of different interventions in the context of pain management paradigms. For instance, the inclusion of lidocaine as a potential therapeutic agent, despite the lack of high-quality randomized controlled trials supporting its use in neuropathic pain, indicates an ongoing exploration of topically applied treatments. This exploration represents an attempt to diversify the arsenal against TN pain, although the evidence to support such a strategy is still emerging. Furthermore, the critical examination of surgical interventions, such as neurectomy, emphasizes the transition to invasive measures when pharmacological management proves insufficient [61]. While these treatments offer a respite in certain cases, their efficacy and utility compared with medical management raise pertinent questions regarding the optimal timing and patient selection for such interventions. The evolving landscape highlights the delicate balance between pharmacological and surgical approaches and argues for a personalized treatment strategy that accommodates individual patient responses and preferences. In conclusion, the management of TN remains a challenging endeavor, characterized by a continuous search for more effective, tolerable, and patient-specific treatment options. The current evidence, which predominantly favors antiepileptic drugs as the cornerstone of pharmacological intervention, necessitates further exploration and substantiation of both existing and emerging therapies. The juxtaposition of traditional pharmacological treatments with novel interventions and surgical options underscores the dynamic nature of TN management and the imperative for ongoing research to refine and expand treatment paradigms [58,60,61] (Table 1).

### 6.3. Neurosurgical Interventions

The investigation of neurosurgical interventions for TN is a critical area of investigation, given the debilitating nature of the condition and the search for efficacious treatments. While the European Academy of Neurology provides guidelines recommending a medical management approach prior to surgery, the lack of sham-controlled or comparative trials of neurosurgical interventions represents a gap in the literature. Microvascular decompression (MVD), a non-destructive procedure, is emerging as a first-choice surgery, with high efficacy reported in pain relief during long-term follow-ups.

The development of MVD began in the early 20th century with Walter Dandy, one of the first surgeons to use a retrocerebellar approach to expose the trigeminal nerve, who made pioneering observations on trigeminal nerve compression [62]. Dandy accidentally performed the first microvascular decompression of the trigeminal nerve root, and his findings laid the foundation for subsequent technical developments. In the 1950s, Palle Taarnhøj performed the first intentional decompression of the trigeminal nerve root, marking the beginning of MVD [62,63]. In the 1960s, James Gardner further confirmed that removing diseased tissue or reducing the pressure on nearby blood vessels can significantly relieve trigeminal neuralgia, and gradually developed a systematic surgical plan for MVD. In the 1990s, Peter Jannetta used microscopy technology to observe the compression phenomenon of the trigeminal nerve root entry area, which finally proved Dandy’s hypothesis and promoted the popularization and standardization of MVD technology [62]. MVD surgical techniques have improved significantly over the past few decades. Early MVD surgeries were mostly used as open surgeries, resulting in large wounds and long recovery periods. With the development of microsurgery technology and changes in concepts, minimally invasive techniques have gradually become popular, allowing surgeons to reduce damage to normal tissues through smaller incisions and more delicate operations. In addition, the application of intraoperative microscopy and neuromonitoring technology allows surgeons to more accurately position and decompress, improving the success rate of surgery [64,65]. At present, the materials that can be used in microvascular decompression surgery mainly include polytetrafluoroethylene (Teflon), sponges (such as gelatin sponges), and other biological materials (such as patches and autologous bones, etc.). Each of these materials has its own unique physical properties and clinical effects [66,67,68].

However, understanding the nuanced outcomes, such as the effectiveness of MVD in different patient demographics and under different clinical conditions, requires a deeper dive into the literature. A central concern in the clinical management of TN is the selection of surgical treatment, where MVD is generally preferred for patients with classic TN due to its high efficacy. This is further accentuated by findings suggesting a notable effectiveness of MVD in patients with classic versus idiopathic TN, although a rigorous comparative analysis based specifically on neuroanatomical changes is lacking in the literature [53]. Such an oversight underscores the need to differentiate treatment efficacy between these TN classifications, thereby refining surgical candidacy criteria. Complications associated with neurosurgical procedures are a critical factor in the decision-making process for both clinicians and patients considering surgery. Although severe complications are rare, the incidence of less serious complications warrants attention and detailed discussion with patients regarding potential outcomes. It is essential to highlight that, while the procedure has high success rates, the inherent risks need to be communicated effectively, taking into account the individual patient context. The recent literature sheds light on other dimensions that influence outcomes after MVD, such as racial disparities in postoperative pain outcomes. Raymond et al. found that TN patients who identified as Black or African American had worse postoperative outcomes compared with White patients [69], highlighting the multifaceted nature of surgical efficacy that transcends mere procedural success to include socioeconomic, genetic, and systemic healthcare access issues. Such findings point to the need for broader more inclusive studies that can account for these disparities and thereby guide more equitable healthcare practices.

The role of neuroimaging, particularly magnetic resonance imaging (MRI), in predicting surgical outcome in TN has been highlighted, suggesting a non-invasive method to generate objective biomarkers of surgical response [53]. This has the potential to streamline the selection process of appropriate surgical candidates, ensuring that interventions are tailored to those most likely to benefit [70]. Despite the robust evidence supporting the efficacy of MVD, the neurosurgical literature on TN remains predominantly retrospective in nature. This retrospective bias implies that reported outcomes and complication rates may not fully reflect the wider patient population due to the lack of independent observer-based evaluations. Consequently, while historical data provide valuable insights, the emerging consensus underscores the urgent need for prospective, controlled, and comparative trials. In summary, while MVD is a cornerstone of the surgical management of TN, demonstrating high efficacy rates and relatively low risk of severe complication, the journey towards optimal patient-specific intervention strategies requires an expanded investigative lens. Addressing the notable gaps—such as the implications of racial disparities on postoperative outcomes [69], the predictive value of MRI in surgical planning [70], and the adversity of prospective trial data—enlightens the path towards more nuanced and equitable neurosurgical care paradigms for TN.

## 7. Looking to the Future: LIPUS Treatment for Neurological Disorders

### 7.1. Promising LIPUS Treatment in Neuropathy (Table 1)

Damage or injury to the peripheral nerves results in neuropathy. Traumatic injury, infection, metabolic issues, hereditary causes, and exposure to toxins can all lead to peripheral neuropathy [71]. Sensory and motor neuropathy are the two primary forms of peripheral neuropathy. Damage to the nerves that affect touch, temperature, pain, and other sensations to the brain is known as sensory neuropathy. Clinically, peripheral sensory neuropathies can cause conditions ranging from excruciating neuropathic disease to complete absence of pain [72]. Damage to the nerves that govern movement is another name for motor neuropathy. Macrotrauma can cause nerves to be stretched, crushed, compressed, or detached from the spinal cord. Examples of these injuries include those caused by sports, medical operations, car accidents, and falls. 

There is increasing evidence that neuropathic pain is modulated by injury-induced neuroinflammation in the central and peripheral nervous systems [73,74]. Pro-inflammatory cytokines are secreted by Schwann cells, which are activated immediately after damage. In the dorsal root ganglion (DRG), inflammatory mediators trigger the immunological response of both damaged and unharmed sensory neurons and the growth of satellite glial cells. Pro-inflammatory cytokines released by immune cells that have been recruited to the DRG also activate DRG neurons and satellite glial cells, creating a crosstalk that prolongs neuropathic pain. Finally, neuroimmune interactions in the central nervous system stimulate microglia. Neuropathic pain eventually results from non-resolving neuroinflammation caused by the cytokine–chemokine network [73,75]. Combined therapies targeting neuroinflammation are thought to be an effective way of treating neuropathic pain [75]. 

There are three categories of potential treatments for neuropathy: minimally invasive, non-invasive, and surgical. Peripheral nerve surgery may be needed to reconstruct or repair damaged nerves if they are severely compressed, sliced, or do not heal on their own. Medication, physiotherapy, or massage therapy are non-surgical treatment alternatives for these minor nerve injuries. A number of treatments, including mesenchymal stem cell therapy, have recently demonstrated great promise in the clinic [76,77]. 

**Figure 1:**
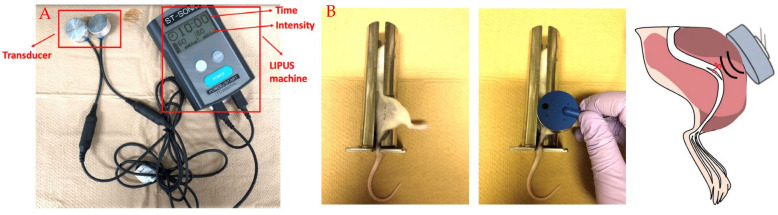
(**A**) Low-intensity pulsed ultrasound machine (ITO Co., Saitama, Japan), (**B**) LIPUS application in partial sciatic nerve ligation model in animal experiments.

This is a minimally invasive technique. Nonetheless, this form of cell administration has certain risks, including arrhythmia, ossification, and calcification [78,79]. The management of neuropathic pain is complicated by the diverse processes behind it and the accompanying psychological and emotional factors associated with chronic pain. The search for clinically viable therapies and medications for neuropathic pain continues, leading to significant interest in anti-inflammatory ultrasound therapy. LIPUS is acknowledged as an effective, diagnostic, therapeutic, and safe tool in medicine. The ultrasound intensity can be set and delivered through an ultrasound probe, which is coupled to the place overlying the injury with a water-based gel [80]. Typical application of the device is for 20 min on a daily basis for the duration of treatment [81] (Figure 1). The patient or the practitioner can apply the machine by themselves, and the cost depends on the country and hospital. A previous study has demonstrated that LIPUS can accelerate sciatic nerve regeneration after neurotomy [82]. LIPUS facilitates spinal fusion by modulating macrophage polarization [83]. Conversely, LIPUS has no adverse effects, including harmful, carcinogenic, or thermal effects that could compromise living tissue. Our previous research has revealed the anti-inflammatory and regenerative properties of LIPUS in several conditions, including sialadenitis, skeletal muscle damage, and knee joint synovitis [84,85,86].

### 7.2. LIPUS Treatment Regenerates Mandibular Nerve

Orofacial surgery, including impacted tooth extraction, dental implant placement, mandibular fracture repair, or whole mandibular reconstruction, may occasionally result in damage to the inferior alveolar nerve, namely the mandibular nerve [87]. The inferior alveolar nerve is the most substantial of the three branches of the trigeminal nerve and has both motor and sensory functions [88]. Bilateral sagittal split ramus osteotomy, the predominant orthognathic surgical intervention to correct mandibular anomalies, frequently leads to neurosensory disturbances in the orofacial area innervated by the inferior alveolar nerve [89].

Numerous studies have shown that LIPUS exposure enhances the regeneration of the inferior alveolar nerve after damage (Table 2). Sato et al. [90] investigated alterations in mechanical sensitivity of the facial skin above the mental foramen following LIPUS therapy of the transected inferior alveolar nerve. LIPUS was administered at a frequency of 1.0 MHz, an intensity of 30 mW/cm^2^, and a pulse rate of 20% (BR-Sonic-Pro, ITO Physiotherapy & Rehabilitation, Saitama, Japan), with daily exposure of the transected inferior alveolar nerve for 20 min over a duration of 28 days after transection. The researchers found that daily exposure to LIPUS promoted both morphological and functional regeneration of the inferior alveolar nerve, indicating that LIPUS is an effective and innovative treatment for mandibular nerve injury. Tsuchimochi et al. [91] investigated the role of neurotrophin-3 (NT-3) in the functional regeneration of the transected inferior alveolar nerve following LIPUS treatment in rats. Given that NT-3 predominantly associates with tropomyosin receptor kinase C (TrkC), the researchers examined the impact of TrkC neutralization on the enhancement of facial mechanosensory disturbance recovery facilitated by LIPUS treatment. LIPUS treatment dramatically improved functional recovery of sensory deficits in facial skin. Schwann cells in the severed inferior alveolar nerve exhibited NT-3 expression, and LIPUS therapy enhanced NT-3 expression. Simultaneously, the persistent TrkC neutralization at the nerve transection site impeded the enhanced recovery from mechanosensory impairment aided by LIPUS therapy [91]. The findings suggest that LIPUS treatment improves the recovery of orofacial mechanosensory function after inferior alveolar nerve transection by promoting NT-3 signaling.

### 7.3. LIPUS Ameliorates Partial Sciatic Nerve Ligation-Induced Neuropatic Pain

The sciatic nerve (SCN) is a mixed nerve responsible for both motor and sensory functions in the lower limbs. It extends from the lower back to the foot. The SCN innervates the muscles of the posterior thigh compartment. The muscles involved are the biceps femoris, semimembranosus, semitendinosus, and the ischial portion of the adductor magnus, which facilitate knee flexion and hip adduction [92]. 

Mice were subjected to peritoneal anesthesia. The ipsilateral sciatic nerves underwent exposure to LIPUS for 20 min per day for 7 days using a circular surface transducer with an area of 5.0 cm^2^. The ultrasound signal was delivered at a spatially averaged intensity of 30 mW/cm^2^ at a frequency of 1.5 MHz. Consequently, mechanical allodynia was significantly induced in all mice by partial sciatic nerve ligation (PSL) and persisted for one week. LIPUS attenuated hypersensitivity compared with the PSL group on day 7. LIPUS exposure was found to increase M2 macrophages in the SCN and reduce PSL-induced inflammation. Following LIPUS treatment, the expression levels of CD206, arginase-1 (Arg-1) and YM-1 were significantly elevated in the SCN. LIPUS decreased the expression of the pan-macrophage marker F4/80, the M1 macrophage marker iNOS, and the pro-inflammatory cytokines TNF-α and IL-1β. The restored hypersensitivity, along with the reduced expression of Iba1 and pro-inflammatory cytokines in the spinal cord of the LIPUS group, suggests that LIPUS exposure may serve as a promising approach for regulating nerve injury-induced neuropathic pain [93].

### 7.4. Possible Mechanisms of Action of LIPUS on Neuropathy

The effects of LIPUS on peripheral nerve regeneration are evident; however, the underlying mechanisms remain unclear. Following peripheral nerve injury, Schwann cells identify axonal damage, attract macrophages from blood vessels to the affected nerves, and generate various inflammatory mediators [93,94,95,96]. Macrophages and Schwann cells function to remove myelin debris and facilitate remodeling of the extracellular matrix.

Macrophage polarization significantly influences the pathogenesis of nerve regeneration. Resident and recruited pro-inflammatory (M1) macrophages function to eliminate cellular debris and exhibit elevated levels of oxidative and tissue remodeling proteins. Macrophages secrete several matrix metalloproteinases (MMPs), including MMP1, 2, 3, 8, 9, 10, 11, 12, 13, and 14. These mediators exacerbate damage in the presence of unresolved inflammation [97]. M1 macrophages influence the microenvironment by producing pro-inflammatory cytokines (IL-1β and TNF-α) and nitric oxide (NO), whereas M2 macrophages counteract this condition. A recent study investigated the anti-inflammatory effects of LIPUS in a spinal fusion model. LIPUS induced the transformation of M1 macrophages into M2 macrophages in vivo [83]. Our recent study on neuropathic pain demonstrated that LIPUS increased M2 macrophages in the sciatic nerve and attenuated ligation-induced severe inflammation [93]. LIPUS reduced the expression of Iba1 and pro-inflammatory cytokines in the central nervous system. LIPUS may represent a potential therapeutic approach for neuropathic pain following injury.

Schwann cells play a vital role in peripheral nerve regeneration and pain modulation. A significant number of studies have investigated the impact of LIPUS on Schwann cells [94,98,99,100]. Application of LIPUS has been shown to facilitate the redifferentiation of repair Schwann cells into myelinating Schwann cells and/or re-myelination [99,100]. Yue et al. [101] showed that the application of LIPUS enhanced the expression of proteins associated with Schwann cell myelination in vitro. This study focused on Schwann cells in culture under non-myelinating conditions, and the in vivo pro-myelinating effect of LIPUS exposure on the myelin sheath is still debated. Numerous studies suggest that the secretion of neurotrophic factors by Schwann cells mediates axon regrowth. Neurotrophic factors facilitate neuroprotection, axonal regrowth, and myelinogenesis following peripheral nerve injury [36]. LIPUS has been proposed to enhance neurotrophic factor secretion in Schwann cells; however, the mechanisms by which LIPUS modulates this secretion following severe nerve injury are not yet fully understood [102,103,104,105]. A recent study has demonstrated that Schwann cells modulate neuropathic pain through the Pannexin 1 (Panx 1) channel [106]. Our results indicate that LIPUS treatment downregulated the Schwann cell Panx 1 channel, thereby alleviating nerve injury-induced trigeminal neuropathic pain.

The alternative hypothesis is the repair and regeneration of injured nerves following LIPUS treatment occurs directly via axons [107,108]. An in vitro study demonstrated that LIPUS exposure of DRG neurons enhanced neurite outgrowth, possibly potentially through the activation of the Netrin-1/DDC pathway [109]. Additionally, it has been proposed that LIPUS facilitates axonal regrowth by reducing the expression of axonal semaphorin 3A, which inhibits axonal regeneration, and by diminishing GSK-3b signaling, a potential barrier to axonal regrowth [105]. After peripheral nerve injury, LIPUS may affect myeloid cells or vascular endothelial cells [110,111]. However, there is limited information on the effect of LIPUS on these cells during the process of peripheral nerve regeneration. 

**Table 2:** Summary of LIPUS studies and their characteristics on neuropathy.

	Animals and Experimental Models	LIPUS Parameters	Time of Stimulation	Major Findings
In vivo Sato et al. [86]	Sprague Dawley rats Inferior alveolar nerve (IAN) injury	Intensity: 30 mW/cm^2^ Pulsed frequency: 1.0 MHz Pulse rate: 20%	20 min/day for 28 days	LIPUS exposure to transected IAN significantly promoted recovery of the head-withdrawal threshold and increased the number of TG neurons.
In vivo Liu et al. [93]	Mice Partial sciatic nerve ligation (PSL)	Intensity: 30 mW/cm^2^ Pulsed frequency: 1.5 MHz Pulse rate: 20%	20 min/day for 7 days	The daily use of LIPUS for 7 days increased the number of M2 macrophages in the sciatic nerve and reduced PSL-induced neuropathic pain.
In vivo Zhang et al. [83]	Sprague Dawley rats Spinal fusion	Intensity: 30 ± 30% mW/cm^2^ Pulsed frequency: 1.5 ± 5% MHz Pulse rate: 200 ± 10% μs	20 min/day for 5 day/week	LIPUS application in spinal fusion model stimulated the early appearance of resident macrophages and promoted bone formation through endochondral ossification.
In vivo Jiang et al. [100]	Sprague Dawley rats Sciatic nerve autograft reversal transplantation surgery	Intensity: 250/500/750 mW/cm^2^ Pulsed frequency: 1.0 KHz Pulse rate: 20%	20 min/day for 7 days	LIPUS at 250 mW/cm^2^ significantly induced faster rate of axonal regeneration.
In vitro Yue et al. [101]	Rat Schwann cell line RSC96	Intensity: 20 mW/cm^2^ Pulsed frequency: 1.0 MHz Pulse rate: 20%	10 min/day for 2/5/8 days	LIPUS may have potential in nerve regeneration with potential clinical relevance.
In vivo Wang et al. [102]	Rats Sciatic nerve crush injury	Intensity: 140 mW/cm^2^ Pulsed frequency: 1.0 MHz Pulse rate: 20%	5 min/day for 14 days and 5 day/week	LIPUS contributed to rapid functional and histologic improvement and upregulated BDNF expression after sciatic nerve crush injury in rats.
In vitro Ren et al. [103]	Sprague Dawley rats Sciatic nerve primary Schwann cells	Intensity: 27.37 mW/cm^2^	10 min/day for 5 days	LIPUS promoted Schwann cell viability and proliferation by increasing Cyclin D1 expression via enhancing the GSK-3β/β-catenin signaling pathway.
In vitro Wen et al. [109]	Rats Primary cortical neurons	Intensity: 0.008/0.12/0.21 W/cm^2^ Pulsed frequency: 1.0 MHz Pulse rate: 20%	5 min/day	LIPUS significantly increased neuronal viability and inhibited neuronal apoptosis in vitro. Also, LIPUS at 0.12 W/cm^2^ enhanced the axonal growth guidance by activation of netrin-1. LIPUS at 0.12 W/cm^2^ promoted the functional restoration of rat injured nerves in vivo.

## 8. Conclusions

The pathophysiological basis of TN, especially the involvement of neurovascular contact, has been better understood, and the clinical aspects of the condition have been much better documented. This development has improved the knowledge of the most typical clinical signs of TN and has drawn attention to the significance of assessing the degree of neurovascular contact prior to surgery. Future research should focus on the pathophysiology of TN, including the processes underlying the marked sex differences in this condition. The development of more efficient and well-tolerated therapies is urgently needed. LIPUS is considered a potential therapeutic option for various diseases. It has an anti-inflammatory effect that promotes the proliferation of M2 macrophages. Mechanical waves of lower energy reduce cytokine production. Additionally, there is growing evidence supporting the potential role of LIPUS in various mechanisms. LIPUS, as a non-invasive treatment method, shows greater potential ability compared with invasive treatments, and is a promising treatment not only for TN but also for neuropathy.

## Figures and Tables

**Table 1:** Results from the treatment of trigeminal neuralgia.

	Medication	Major Findings
Muñoz-Vendrell et al. [54]	Intravenous lacosamide and phenytoin injection	Intravenous lacosamide and phenytoin can be effective and safe treatments for acute pain in TN, suggesting that lacosamide may be better tolerated than phenytoin and lead to lower readmission rates and sustained pain relief.
Zhou et al. [55]	Lidocaine aerosol sprayed onto the oral and/or nasal mucosa in patients suffering from acute TN exacerbations	The study suggests that this approach may be a promising treatment option, consistent with the clinical view that lidocaine injections into trigger areas may provide short-term relief.
Kolakowski Lukasz et al. [57]	Fosphenytoin	Fosphenytoin shows safety and effectiveness in relieving acute trigeminal neuralgia.
Rozen et al. [58] and Skoglund et al. [59]	Carbamazepine and oxcarbazepine	Carbamazepine and oxcarbazepine have demonstrated efficacy in reducing the neuropathic pain associated with TN; however, their use is often limited by adverse side effects.
Skoglund et al. [59] and Derry et al. [60]	Gabapentin and nortriptyline	Gabapentin and nortriptyline represent alternatives for carbamazepine and oxcarbazepine, although their comparative efficacy and acceptability require further clarification through rigorous evaluation.
Skoglund et al. [59]	Botulinum toxin type A	Botulinum toxin type A as an adjunctive or alternative treatment modality signals a progressive shift towards exploring non-traditional pharmacological interventions for TN.

## References

[1] McMillan R. (2011). Trigeminal Neuralgia—A Debilitating Facial Pain. Rev. Pain..

[2] Puskar K.R., Droppa M. (2015). Trigeminal neuralgia: Pain, pricks, and anxiety. J. Gerontol. Nurs..

[3] Anwar H.A., Ramya Krishna M., Sadiq S., Ramesh Kumar R., Venkatarathnam V., Saikiran G. (2022). A study to evaluate neurovascular conflict of trigeminal nerve in trigeminal neuralgia patients with the help of 1.5 T MR imaging. Egypt. J. Radiol. Nucl. Med..

[4] Zhao W., Yin C., Ma L., Ding M., Kong W., Wang Y. (2024). Predictive value of MRI for identifying symptomatic neurovascular compressions in classical trigeminal neuralgia: A PRISMA-compliant meta-analysis. BMC Neurol..

[5] Bora N., Parihar P., Raj N., Shetty N., Nunna B. (2023). A Systematic Review of the Role of Magnetic Resonance Imaging in the Diagnosis and Detection of Neurovascular Conflict in Patients With Trigeminal Neuralgia. Cureus.

[6] Chai W., You C., Zhang W., Peng W., Tan L., Guan Y., Chen K. (2019). Diffusion tensor imaging of microstructural alterations in the trigeminal nerve due to neurovascular contact/compression. Acta Neurochir..

[7] Sessle B.J. (2021). Chronic Orofacial Pain: Models, Mechanisms, and Genetic and Related Environmental Influences. Int. J. Mol. Sci..

[8] Mannerak M.A., Lashkarivand A., Eide P.K. (2021). Trigeminal neuralgia and genetics: A systematic review. Mol. Pain.

[9] Wei X., Zhou H., Zhang S., Hu X., Wei Z., Li Y. (2023). A comprehensive two-sample Mendelian randomization analysis of trigeminal neuralgia and modifiable risk factors. Front. Neurol..

[10] Li J., Wang W., Huang W., Lin J., Zheng X., Zhang M., Lin Z. (2024). Effect of Percutaneous Balloon Compression on Trigeminal Neuralgia and Clinical Significance of NLRP3 Before and After Treatment. Altern. Ther. Health Med..

[11] O’Callaghan L., Floden L., Vinikoor-Imler L., Symonds T., Giblin K., Hartford C., Zakzewska J.M. (2020). Burden of illness of trigeminal neuralgia among patients managed in a specialist center in England. J. Headache Pain.

[12] Xin Z., Lin G., Lei H., Lue T.F., Guo Y. (2016). Clinical applications of low-intensity pulsed ultrasound and its potential role in urology. Transl. Androl. Urol..

[13] Kusuyama J., Bandow K., Shamoto M., Kakimoto K., Ohnishi T., Matsuguchi T. (2014). Low intensity pulsed ultrasound (LIPUS) influences the multilineage differentiation of mesenchymal stem and progenitor cell lines through ROCK-Cot/Tpl2-MEK-ERK signaling pathway. J. Biol. Chem..

[14] Sandel T., Eide P.K. (2013). Long-term results of microvascular decompression for trigeminal neuralgia and hemifacial spasms according to preoperative symptomatology. Acta Neurochir..

[15] Mistry A.M., Niesner K.J., Lake W.B., Forbes J.A., Shannon C.N., Kasl R.A., Konrad P.E., Neimat J.S. (2016). Neurovascular Compression at the Root Entry Zone Correlates with Trigeminal Neuralgia and Early Microvascular Decompression Outcome. World Neurosurg..

[16] Di Stefano G., Yuan J.H., Cruccu G., Waxman S.G., Dib-Hajj S.D., Truini A. (2020). Familial trigeminal neuralgia—A systematic clinical study with a genomic screen of the neuronal electrogenisome. Cephalalgia.

[17] Peker S., Kurtkaya O., Uzun I., Pamir M.N. (2006). Microanatomy of the central myelin-peripheral myelin transition zone of the trigeminal nerve. Neurosurgery.

[18] Rappaport Z.H., Govrin-Lippmann R., Devor M. (1997). An electron-microscopic analysis of biopsy samples of the trigeminal root taken during microvascular decompressive surgery. Stereotact. Funct. Neurosurg..

[19] Love S., Coakham H.B. (2001). Trigeminal neuralgia: Pathology and pathogenesis. Brain.

[20] Devor M., Amir R., Rappaport Z.H. (2002). Pathophysiology of trigeminal neuralgia: The ignition hypothesis. Clin. J. Pain..

[21] Magerl W., Treede R.D. (2004). Secondary tactile hypoesthesia: A novel type of pain-induced somatosensory plasticity in human subjects. Neurosci. Lett..

[22] Miles J.B., Eldridge P.R., Haggett C.E., Bowsher D. (1997). Sensory effects of microvascular decompression in trigeminal neuralgia. J. Neurosurg..

[23] Leandri M., Eldridge P., Miles J. (1998). Recovery of nerve conduction following microvascular decompression for trigeminal neuralgia. Neurology.

[24] Tohyama S., Hung P.S., Cheng J.C., Zhang J.Y., Halawani A., Mikulis D.J., Hodaie M. (2020). Trigeminal neuralgia associated with a solitary pontine lesion: Clinical and neuroimaging definition of a new syndrome. Pain.

[25] Tanaka B.S., Zhao P., Dib-Hajj F.B., Morisset V., Tate S., Waxman S.G., Dib-Hajj S.D. (2016). A gain-of-function mutation in Nav1.6 in a case of trigeminal neuralgia. Mol. Med..

[26] Siqueira S.R., Alves B., Malpartida H.M., Teixeira M.J., Siqueira J.T. (2009). Abnormal expression of voltage-gated sodium channels Nav1.7, Nav1.3 and Nav1.8 in trigeminal neuralgia. Neuroscience.

[27] Ericson H., Abu Hamdeh S., Freyhult E., Stiger F., Backryd E., Svenningsson A., Gordh T., Kultima K. (2019). Cerebrospinal fluid biomarkers of inflammation in trigeminal neuralgia patients operated with microvascular decompression. Pain.

[28] DeSouza D.D., Hodaie M., Davis K.D. (2014). Abnormal trigeminal nerve microstructure and brain white matter in idiopathic trigeminal neuralgia. Pain.

[29] Arrese I., Lagares A., Alday R., Ramos A., Rivas J.J., Lobato R.D. (2008). Typical trigeminal neuralgia associated with brainstem white matter lesions on MRI in patients without criteria of multiple sclerosis. Acta Neurochir..

[30] Obermann M., Yoon M.S., Ese D., Maschke M., Kaube H., Diener H.C., Katsarava Z. (2007). Impaired trigeminal nociceptive processing in patients with trigeminal neuralgia. Neurology.

[31] Leonard G., Goffaux P., Mathieu D., Blanchard J., Kenny B., Marchand S. (2009). Evidence of descending inhibition deficits in atypical but not classical trigeminal neuralgia. Pain.

[32] Younis S., Maarbjerg S., Reimer M., Wolfram F., Olesen J., Baron R., Bendtsen L. (2016). Quantitative sensory testing in classical trigeminal neuralgia-a blinded study in patients with and without concomitant persistent pain. Pain.

[33] Ibrahim S. (2014). Trigeminal neuralgia: Diagnostic criteria, clinical aspects and treatment outcomes. A retrospective study. Gerodontology.

[34] Koh W., Lim H., Chen X. (2021). Atypical triggers in trigeminal neuralgia: The role of A-delta sensory afferents in food and weather triggers. Korean J. Pain.

[35] Zoller S., Oertel M.F., Stieglitz L.H. (2022). Trigeminal Neuralgia—What Do We Know about the Causes, Diagnosis and Treatment?. Praxis.

[36] Ozlem K.K., Alpay A., Mustafa A.H., Gizem T., Bahar A.B., Mehmet B. (2018). T2 Signal Intensity of the Trigeminal Nerve: A New Diagnostic Criteria for Trigeminal Neuralgia?. Curr. Med. Imaging.

[37] Slettebo H. (2021). Is this really trigeminal neuralgia? Diagnostic re-evaluation of patients referred for neurosurgery. Scand. J. Pain.

[38] Yeh K.Y., Chiu H.W., Tseng W.T., Chen H.C., Yen C.T., Lu S.S., Lin M.L. (2021). A Dual-Mode Multifunctional Pulsed Radio-Frequency Stimulator for Trigeminal Neuralgia Relief and its Animal Model. IEEE Trans. Biomed. Circuits Syst..

[39] Kalluri A.L., So R.J., Ran K.R., Xie M.E., Kilgore C., Nair S.K., Huang J., Bettegowda C., Xu R. (2023). Preoperative Characteristics and Postoperative Pain Outcomes in Trigeminal Neuralgia With Concomitant Autoimmune Disease. Neurosurgery.

[40] Cruccu G., Finnerup N.B., Jensen T.S., Scholz J., Sindou M., Svensson P., Treede R.D., Zakrzewska J.M., Nurmikko T. (2016). Trigeminal neuralgia: New classification and diagnostic grading for practice and research. Neurology.

[41] Benoliel R., Svensson P., Evers S., Wang S.J., Barke A., Korwisi B., Rief W., Treede R.D., IASP Taskforce for Classification of Chronic Pain (2019). The IASP classification of chronic pain for ICD-11: Chronic secondary headache or orofacial pain. Pain.

[42] Bendtsen L., Zakrzewska J.M., Heinskou T.B., Hodaie M., Leal P.R.L., Nurmikko T., Obermann M., Cruccu G., Maarbjerg S. (2020). Advances in diagnosis, classification, pathophysiology, and management of trigeminal neuralgia. Lancet Neurol..

[43] Oleson J. (2018). 13.1.1 Trigeminal neuralgia. The International Classification of Headache Disorders. 3rd Edition. https://ichd-3.org/13-painful-cranial-neuropathies-and-other-facial-pains/.

[44] Jurge S. (2016). Pain part 7: Trigeminal neuralgia. Dent. Update.

[45] De Stefano G., Litewczuk D., Mollica C., Di Pietro G., Galosi E., Leone C., Falco P., Tullo M.G., Caramia F., Truini A. (2023). Sex differences in trigeminal neuralgia: A focus on radiological and clinical characteristics. Neurol. Sci..

[46] Cruccu G., Di Stefano G., Truini A. (2020). Trigeminal Neuralgia. N. Engl. J. Med..

[47] Bendtsen L., Birk S., Kasch H., Aegidius K., Sorensen P.S., Thomsen L.L., Poulsen L., Rasmussen M.J., Kruuse C., Jensen R. (2012). Reference programme: Diagnosis and treatment of headache disorders and facial pain. Danish Headache Society, 2nd Edition, 2012. J. Headache Pain.

[48] Maarbjerg S., Gozalov A., Olesen J., Bendtsen L. (2014). Trigeminal neuralgia—A prospective systematic study of clinical characteristics in 158 patients. Headache.

[49] Siviero M., Alvarez F.K., Okada M., Teixeira M.J., de Siqueira J.T., de Siqueira S.R. (2011). Facial sensibility of patients with trigeminal neuralgias. Clin. Neurol. Neurosurg..

[50] Maier C., Baron R., Tolle T.R., Binder A., Birbaumer N., Birklein F., Gierthmühlen J., Flor H., Geber C., Huge V. (2010). Quantitative sensory testing in the German Research Network on Neuropathic Pain (DFNS): Somatosensory abnormalities in 1236 patients with different neuropathic pain syndromes. Pain.

[51] Hegarty A.M., Zakrzewska J.M. (2011). Differential diagnosis for orofacial pain, including sinusitis, TMD, trigeminal neuralgia. Dent. Update.

[52] Moore D., Chong M.S., Shetty A., Zakrzewska J.M. (2019). A systematic review of rescue analgesic strategies in acute exacerbations of primary trigeminal neuralgia. Br. J. Anaesth..

[53] Bendtsen L., Zakrzewska J.M., Abbott J., Braschinsky M., Di Stefano G., Donnet A., Eide P.K., Leal P.R., Maarbjerg S., May A. (2019). European Academy of Neurology guideline on trigeminal neuralgia. Eur. J. Neurol..

[54] Munoz-Vendrell A., Teixidor S., Sala-Padro J., Campoy S., Huerta-Villanueva M. (2022). Intravenous lacosamide and phenytoin for the treatment of acute exacerbations of trigeminal neuralgia: A retrospective analysis of 144 cases. Cephalalgia.

[55] Zhou X., Shen Y., Zhao C., Luo F. (2023). Lidocaine aerosol sprayed on oral and/or nasal mucosa for the rescue of acute trigeminal neuralgia exacerbations: A retrospective study. Cephalalgia.

[56] Baydoun J., Lin A., Miya J. (2023). Low-dose Fosphenytoin for Aborting Acute Trigeminal Neuralgia Pain: A Case Report. Clin. Pract. Cases Emerg. Med..

[57] Kolakwowski L., Pohl H., Kleinsorge M.T., Wegener S. (2024). Phenytoin relieves acute exacerbations of trigeminal neuralgia: Results of a retrospective case series. Cephalalgia Rep..

[58] Rozen T.D. (2001). Antiepileptic drugs in the management of cluster headache and trigeminal neuralgia. Headache.

[59] Skoglund L.A. (2000). Anticonvulsants are effective for acute pain in trigeminal neuralgia. Evid. Based Dent..

[60] Derry S., Wiffen P.J., Aldington D., Moore R.A. (2015). Nortriptyline for neuropathic pain in adults. Cochrane Database Syst. Rev..

[61] Derry S., Wiffen P.J., Moore R.A., Quinlan J. (2014). Topical lidocaine for neuropathic pain in adults. Cochrane Database Syst. Rev..

[62] Patel S.K., Markosian C., Choudhry O.J., Keller J.T., Liu J.K. (2020). The historical evolution of microvascular decompression for trigeminal neuralgia: From Dandy’s discovery to Jannetta’s legacy. Acta Neurochir..

[63] Huang C.I., Chen I.H., Lee L.S. (1992). Microvascular decompression for hemifacial spasm: Analyses of operative findings and results in 310 patients. Neurosurgery.

[64] Heuser K., Kerty E., Eide P.K., Cvancarova M., Dietrichs E. (2007). Microvascular decompression for hemifacial spasm: Postoperative neurologic follow-up and evaluation of life quality. Eur. J. Neurol..

[65] Engh J.A., Horowitz M., Burkhart L., Chang Y.F., Kassam A. (2005). Repeat microvascular decompression for hemifacial spasm. J. Neurol. Neurosurg. Psychiatry.

[66] Xue F., Shen Z., Wang Y., Kwok S.C., Yin J. (2021). Microvascular decompression for hemifacial spasm involving the vertebral artery: A modified effective technique using a gelatin sponge with a FuAiLe medical adhesive. CNS Neurosci. Ther..

[67] Pressman E., Jha R.T., Zavadskiy G., Kumar J.I., van Loveren H., van Gompel J.J., Agazzi S. (2020). Teflon or Ivalon(R): A scoping review of implants used in microvascular decompression for trigeminal neuralgia. Neurosurg. Rev..

[68] Liu W., Yuan Y., Xiong N., Wang Q., Zhang F., Zhao H., Xu H., Nayaz A., Hendrik P., Sean D.J. (2021). Reconstruction of Craniectomy for Microvascular Decompression with Autologous Particulate Bone. J. Neurol. Surg. Part A Cent. Eur. Neurosurg..

[69] So R.J., Kalluri A.L., Storm K., Nair S.K., Budihal B.R., Huang J., Lim M., Bettegowda C., Xu R. (2023). A racial analysis of pain outcomes following microvascular decompression for trigeminal neuralgia. J. Neurosurg..

[70] Wang Z., Zhao Z., Song Z., Wang Y., Zhao Z. (2022). The application of magnetic resonance imaging (MRI) for the prediction of surgical outcomes in trigeminal neuralgia. Postgrad. Med..

[71] Ashraff S., Siddiqui M.A., Santos D., Carline T. (2019). Prediction of stump healing in lower limb amputation: A narrative review. J. Wound Care.

[72] Kalia A.K., Rosseler C., Granja-Vazquez R., Ahmad A., Pancrazio J.J., Neureiter A., Zhang M., Sauter D., Vetter I., Andersson A. (2024). How to differentiate induced pluripotent stem cells into sensory neurons for disease modelling: A functional assessment. Stem Cell Res. Ther..

[73] Calvo M., Dawes J.M., Bennett D.L. (2012). The role of the immune system in the generation of neuropathic pain. Lancet Neurol..

[74] Ren K., Dubner R. (2010). Interactions between the immune and nervous systems in pain. Nat. Med..

[75] Ji R.R., Chamessian A., Zhang Y.Q. (2016). Pain regulation by non-neuronal cells and inflammation. Science.

[76] Venturi M., Boccasanta P., Lombardi B., Brambilla M., Contessini Avesani E., Vergani C. (2015). Pudendal Neuralgia: A New Option for Treatment? Preliminary Results on Feasibility and Efficacy. Pain Med..

[77] Vickers E.R., Karsten E., Flood J., Lilischkis R. (2014). A preliminary report on stem cell therapy for neuropathic pain in humans. J. Pain Res..

[78] Breitbach M., Bostani T., Roell W., Xia Y., Dewald O., Nygren J.M., Fries J.W., Tiemann K., Bohlen H., Hescheler J. (2007). Potential risks of bone marrow cell transplantation into infarcted hearts. Blood.

[79] Chang M.G., Tung L., Sekar R.B., Chang C.Y., Cysyk J., Dong P., Marban E., Abraham M.R. (2006). Proarrhythmic potential of mesenchymal stem cell transplantation revealed in an in vitro coculture model. Circulation.

[80] Berber R., Aziz S., Simkins J., Lin S.S., Mangwani J. (2020). Low Intensity Pulsed Ultrasound Therapy (LIPUS): A review of evidence and potential applications in diabetics. J. Clin. Orthop. Trauma.

[81] Busse J.W., Morton E., Lacchetti C., Guyatt G.H., Bhandari M. (2008). Current management of tibial shaft fractures: A survey of 450 Canadian orthopedic trauma surgeons. Acta Orthop..

[82] Crisci A.R., Ferreira A.L. (2002). Low-intensity pulsed ultrasound accelerates the regeneration of the sciatic nerve after neurotomy in rats. Ultrasound Med. Biol..

[83] Zhang Z.C., Yang Y.L., Li B., Hu X.C., Xu S., Wang F., Li M., Zhou X.Y., Wei X.Z. (2019). Low-intensity pulsed ultrasound promotes spinal fusion by regulating macrophage polarization. Biomed. Pharmacother..

[84] Nagata K., Nakamura T., Fujihara S., Tanaka E. (2013). Ultrasound modulates the inflammatory response and promotes muscle regeneration in injured muscles. Ann. Biomed. Eng..

[85] Nakamura T., Fujihara S., Yamamoto-Nagata K., Katsura T., Inubushi T., Tanaka E. (2011). Low-intensity pulsed ultrasound reduces the inflammatory activity of synovitis. Ann. Biomed. Eng..

[86] Sato M., Kuroda S., Mansjur K.Q., Khaliunaa G., Nagata K., Horiuchi S., Inubushi T., Yamamura Y., Azuma M., Tanaka E. (2015). Low-intensity pulsed ultrasound rescues insufficient salivary secretion in autoimmune sialadenitis. Arthritis Res. Ther..

[87] Juodzbalys G., Wang H.L., Sabalys G. (2011). Injury of the Inferior Alveolar Nerve during Implant Placement: A Literature Review. J. Oral Maxillofac. Res..

[88] Ghatak R.N., Helwany M., Ginglen J.G. (2024). Anatomy, Head and Neck, Mandibular Nerve.

[89] Roychoudhury S., Nagori S.A., Roychoudhury A. (2015). Neurosensory disturbance after bilateral sagittal split osteotomy: A retrospective study. J. Oral Biol. Craniofac. Res..

[90] Sato M., Motoyoshi M., Shinoda M., Iwata K., Shimizu N. (2016). Low-intensity pulsed ultrasound accelerates nerve regeneration following inferior alveolar nerve transection in rats. Eur. J. Oral Sci..

[91] Tsuchimochi A., Endo C., Motoyoshi M., Tamura M., Hitomi S., Hayashi Y., Shinoda M. (2021). Effect of low-intensity pulsed ultrasound on orofacial sensory disturbance following inferior alveolar nerve injury: Role of neurotrophin-3 signaling. Eur. J. Oral Sci..

[92] Giuffre B.A., Black A.C., Jeanmonod R. (2024). Anatomy, Sciatic Nerve.

[93] Liu Y., Xia L., Kano F., Hashimoto N., Matsuka Y., Yamamoto A., Tanaka E. (2021). Low-Intensity Pulsed Ultrasound Ameliorates Neuropathic Pain Induced by Partial Sciatic Nerve Ligation Via Regulating Macrophage Polarization. J. Oral Health Biosci..

[94] Ronchi G., Nicolino S., Raimondo S., Tos P., Battiston B., Papalia I., Varejão A.S., Giacobini-Robecchi M.G., Perroteau I., Geuna S. (2009). Functional and morphological assessment of a standardized crush injury of the rat median nerve. J. Neurosci. Methods.

[95] Reichert F., Saada A., Rotshenker S. (1994). Peripheral nerve injury induces Schwann cells to express two macrophage phenotypes: Phagocytosis and the galactose-specific lectin MAC-2. J. Neurosci..

[96] Peng D.Y., Reed-Maldonado A.B., Lin G.T., Xia S.J., Lue T.F. (2020). Low-intensity pulsed ultrasound for regenerating peripheral nerves: Potential for penile nerve. Asian J. Androl..

[97] Sheu M.L., Pan L.Y., Yang C.N., Sheehan J., Pan L.Y., You W.C., Wang C.C., Pan H.C. (2023). Thrombin-Induced Microglia Activation Modulated through Aryl Hydrocarbon Receptors. Int. J. Mol. Sci..

[98] Vrbova G., Mehra N., Shanmuganathan H., Tyreman N., Schachner M., Gordon T. (2009). Chemical communication between regenerating motor axons and Schwann cells in the growth pathway. Eur. J. Neurosci..

[99] Ni X.J., Wang X.D., Zhao Y.H., Sun H.L., Hu Y.M., Yao J., Wang Y. (2017). The Effect of Low-Intensity Ultrasound on Brain-Derived Neurotropic Factor Expression in a Rat Sciatic Nerve Crushed Injury Model. Ultrasound Med. Biol..

[100] Jiang W., Wang Y., Tang J., Peng J., Wang Y., Guo Q., Li P., Xiao B., Zhang J. (2016). Low-intensity pulsed ultrasound treatment improved the rate of autograft peripheral nerve regeneration in rat. Sci. Rep..

[101] Yue Y., Yang X., Zhang L., Xiao X., Nabar N.R., Lin Y., Hao L., Zhang D., Huo J., Li J. (2016). Low-intensity pulsed ultrasound upregulates pro-myelination indicators of Schwann cells enhanced by co-culture with adipose-derived stem cells. Cell Prolif..

[102] Wang T., Ito A., Xu S., Kawai H., Kuroki H., Aoyama T. (2021). Low-Intensity Pulsed Ultrasound Prompts Both Functional and Histologic Improvements While Upregulating the Brain-Derived Neurotrophic Factor Expression after Sciatic Crush Injury in Rats. Ultrasound Med. Biol..

[103] Ren C., Chen X., Du N., Geng S., Hu Y., Liu X., Wu X., Lin Y., Bai X., Yin W. (2018). Low-intensity pulsed ultrasound promotes Schwann cell viability and proliferation via the GSK-3beta/beta-catenin signaling pathway. Int. J. Biol. Sci..

[104] Jahromy F.Z., Behnam H., Mansoori K., Rahimi A.A., Edalat R., Mobarake J.I. (2013). The effect of ultrasound on the expression of CNTF gene, a possible cause of ultrasound influence on the rate of injured peripheral nerve regeneration. Australas. Phys. Eng. Sci. Med..

[105] Ito A., Wang T., Nakahara R., Kawai H., Nishitani K., Aoyama T., Kuroki H. (2020). Ultrasound therapy with optimal intensity facilitates peripheral nerve regeneration in rats through suppression of pro-inflammatory and nerve growth inhibitor gene expression. PLoS ONE.

[106] Wang Q., Li H.Y., Ling Z.M., Chen G., Wei Z.Y. (2022). Inhibition of Schwann cell pannexin 1 attenuates neuropathic pain through the suppression of inflammatory responses. J. Neuroinflammation.

[107] Ventre D.M., Cluff A., Gagnon C., Diaz Vera D., Koppes R.A., Koppes A.N. (2021). The effects of low intensity focused ultrasonic stimulation on dorsal root ganglion neurons and Schwann cells in vitro. J. Neurosci. Res..

[108] Ventre D., Puzan M., Ashbolt E., Koppes A. (2018). Enhanced total neurite outgrowth and secondary branching in dorsal root ganglion neurons elicited by low intensity pulsed ultrasound. J. Neural. Eng..

[109] Wen J., Deng X., Huang C., An Z., Liu M. (2021). Low-Intensity Pulsed Ultrasound Enhanced Neurite Guidance Growth through Netrin-1/DCC Signal Pathway in Primary Cultured Cortical Neurons of Rats. ACS Chem. Neurosci..

[110] Xu M., Wang L., Wu S., Dong Y., Chen X., Wang S., Li X., Zou C. (2021). Review on experimental study and clinical application of low-intensity pulsed ultrasound in inflammation. Quant. Imaging Med. Surg..

[111] de Lucas B., Perez L.M., Bernal A., Galvez B.G. (2020). Ultrasound Therapy: Experiences and Perspectives for Regenerative Medicine. Genes.

